# The effect of exenatide on fasting bile acids in newly diagnosed type 2 diabetes mellitus patients, a pilot study

**DOI:** 10.1186/s40360-020-00422-5

**Published:** 2020-06-15

**Authors:** Boyu Li, Yanjin Hu, Guang Wang, Lihong Liu

**Affiliations:** 1 Department of Pharmacy, Beijing Chao-Yang Hospital, Capital Medical University, 8 Gongtinan Road, Chaoyang District, Beijing, 100020 China; 2 Department of Endocrinology, Beijing Chao-Yang Hospital, Capital Medical University, 8 Gongtinan Road, Chaoyang District, Beijing, 100020 China

**Keywords:** Exenatide, Type 2 diabetes mellitus, Bile acids, Glycemic control

## Abstract

**Background:**

Glucagon-like peptide-1 receptor agonists (GLP-1 RAs) demonstrated good glycemic efficacy in patients with type 2 diabetes mellitus (T2DM) recent years, whereas studies on GLP-1 RAs’ biliary effects were limited. Therefore, we aimed to assess the effect of exenatide on bile acids (BAs) and investigate the role of BAs in the glycemic control effect of exenatide.

**Methods:**

Thirty-eight newly diagnosed T2DM participants without glucose-lowering drugs intake were recruited. Plasma total bile acids in fasting state (FTBAs) and other parameters were tested at baseline. Then exenatide were applied to the T2DM participants for 12 weeks. FTBAs and glycemic parameters were measured again after exenatide treatment, and correlation analysis between changes of FTBAs and glycemic parameters were conducted to investigate the role of BAs in the glycemic control effect of exenatide.

**Results:**

The baseline FTBAs level of T2DM patients had no significance (3.84 ± 2.06 vs. 3.87 ± 2.89, *P* = 0.954) compared with healthy subjects. After 12-week exenatide treatment for the T2DM patients, FTBAs were decreased from 3.84 ± 2.06 μmol/L to 3.06 ± 1.27 μmol/L (*P* < 0.01). The correlation analysis showed that changes of FTBAs was positively correlated with changes of FPG (*r* = 0.355, *P* < 0.05).

**Conclusions:**

Our results demonstrated a decreased FTBAs level after exenatide treatment for 12 weeks, without the interference of metformin and other glucose-lowering drugs. The reduction of FTBAs might not exert a positive role in the glycemic control effect of exenatide.

**Trial registration:**

Trial registration number: NCT04303819. Registered in March 11, 2020 - Retrospectively registered.

## Background

Glucagon-like peptide-1 receptor agonists (GLP-1 RAs) demonstrated good glycemic efficacy in type 2 diabetes mellitus (T2DM) patients recent years [1]. This class of drugs exploited the insulinotropic and glucagonostatic effects of GLP-1, thereby effectively lowering blood glucose [2]. In addition, GLP-1 (receptor agonists) inhibits gastric emptying, gastric acid secretion and other aspects of proximal gastrointestinal physiology, for example its role in biliary physiology [2].

Bile acids (BAs) are synthesized in liver from cholesterol and released in the intestinal lumen upon food intake [3]. The physiology of BAs was traditionally considered related to its contribution to the assimilation of fat and fat-soluble vitamins [4, 5]. Recent research suggested that BAs also functioned as a metabolic integrator in regulation of glucose metabolism [6–10] and appetite [11], through binding to the nuclear hormone farnesoid X receptor (FXR) and Takeda G protein receptor 5 (TGR5) in multiple organs, leading to regulation of intestinal incretin secretion, hepatic gluconeogenesis, glycogen synthesis and energy expenditure [12]. Thus, BAs must have a role in the glycemic control effect of GLP-1 RAs.

Studies on GLP-1 RAs’ biliary effects were limited and sometimes controversial. Acute administration of exenatide markedly reduced cholecystokinin stimulated gallbladder emptying by 40% in healthy volunteers [13]. Whereas, there was another study demonstrated that liraglutide increased serum levels of deoxycholic acid in the fasting state and postprandial state in T2DM patients [14]. Therefore, we aimed to access the effect of exenatide on BAs and investigate the role of BAs in the glycemic control effect of exenatide.

## Methods

### Participants

Thirty-eight newly diagnosed T2DM patients and 38 healthy subjects with matched BMI were recruited from the endocrinology department of the Beijing Chao-Yang Hospital between January 2014 and January 2015, following the methods of G. Wang et al. 2016 [15]. The diagnostic criteria of T2DM were in accordance with the World Health Organization criteria of 1999 [16]. T2DM patients were excluded if they had a history of hepatobiliary or pancreatic diseases, a history of glucose-lowering drugs intake, change of anti-dyslipidemia therapy regimen, an estimated glomerular filtration rate < 60 mL/min/1.73 m^2^, pregnancy or alcohol intake > 3 units/d. All subjects gave their written, informed consent to participate.

### Intervention

Thirty-eight newly diagnosed T2DM patients received 12 weeks of exenatide injection, 5 μg twice daily by subcutaneous injection for 4 weeks, followed by 10 μg twice daily for 8 weeks, as recommended by the drug manufacturer. Other than that, they didn’t take any other glucose-lowering drugs.

### Laboratory measurements

Total cholesterol (TC), low-density lipoprotein cholesterol (LDL-C), high-density lipoprotein cholesterol (HDL-C), triglycerides (TG), fasting plasma glucose (FPG) and FTBAs were tested using Dimension RxL auto analyzer (Dade Behring Diagnostics, Deer-field, IL, USA). Glycated hemoglobin A1c (HbA1c) was estimated by high-performance liquid chromatography using HLC-723G7 analyzer (Tosoh Corporation, Tokyo, Japan). Fasting serum insulin (FINS) and C-peptide were measured by Access 2 immunoassay system (Beckman Coulter, Inc., Brea, CA, USA). FTBAs was measured by Total Bile Acids Kit (Enzymatic Cycling assay) (Biosino Bio-Technology and science Inc., Beijing, China). Homoeostasis model assessment for insulin resistance (HOMA-IR) = FPG (mmol/L) × FINS(mU/L)/22.5, and homeostasis model assessment β (HOMA-B) was used to estimate β-cell function and calculated by HOMA-B = 20 × FINS (mU/L) / [FPG (mmol/L) − 3.5] [17]. Changes in parameters were expressed as delta parameters, Δ = parameter (after exenatide treatment) − parameter (pre-treatment).

### Statistical analysis

Variables which were normally distributed, such as age, BMI, TC, LDL-C, HDL-C, glucose, FINS, C-peptide, HbA1c and FTBAs, were analyzed by Student’s t test and expressed as means ± SD. Variables which were not normally distributed, such as TG, HOMA-IR and HOMA-B, were analyzed by the Mann–Whitney U test or Wilcoxon test, and expressed as medians (interquartile range, IQR). Correlations between changes of FTBAs and glycemic parameters were measured using the Pearson correlation technique. All analyses were performed using SPSS 22.0 (IBM SPSS Inc., Chicago, IL, USA), and a two-side *P-*value < 0.05 was considered statistically significant.

## Results

### Baseline characteristics

Baseline characteristics were presented in Table 1. T2DM patients and the healthy subjects had no significance in age and BMI. There was difference in LDL, TG and FPG due to the diabetic pathological status. We measured FTBAs and found no difference (T2DM patients vs. healthy subjects, 3.84 ± 2.06 vs. 3.87 ± 2.89, *P* = 0.954).

**Table 1:** Baseline characteristics

Parameters	T2DM patients	Healthy subjects	*P*-value^a^
	(*n* = 38)	(*n* = 38)	
Age, years	48.2 ± 9.0	52.9 ± 15.7	0.074
Male, %	17, 44.7%	18, 47.4%	
Height, cm	170.7 ± 9.4	171.7 ± 6.02	0.572
Weight, kg	93.1 ± 17.1	90.1 ± 8.3	0.722
BMI, kg/m^2^	31.2 (28.5, 35.1)	30.8 (27.7, 33.9)	0.684
TC, mmol/L	5.05 ± 1.11	4.09 ± 0.77	<0.01
HDL-C, mmol/L	1.15 ± 0.46	1.14 ± 0.30	0.995
LDL-C, mmol/L	2.92 ± 0.80	2.23 ± 0.54	<0.01
TG, mmol/L	2.16 (1.26, 4.21)	1.30 (0.87, 2.07)	<0.01
FPG, mmol/L	9.19 ± 3.46	4.41 ± 0.95	<0.01
FTBAs, μmol/L	3.84 ± 2.06	3.87 ± 2.89	0.954

Data presented as means ± SD or medians (interquartile range)

*P* value was calculated by independent sample T test

^a^T2DM patients vs. healthy subjects

### Changes of FTBAs and glycemic parameters after 12-week exenatide treatment

**Fig. 1:**
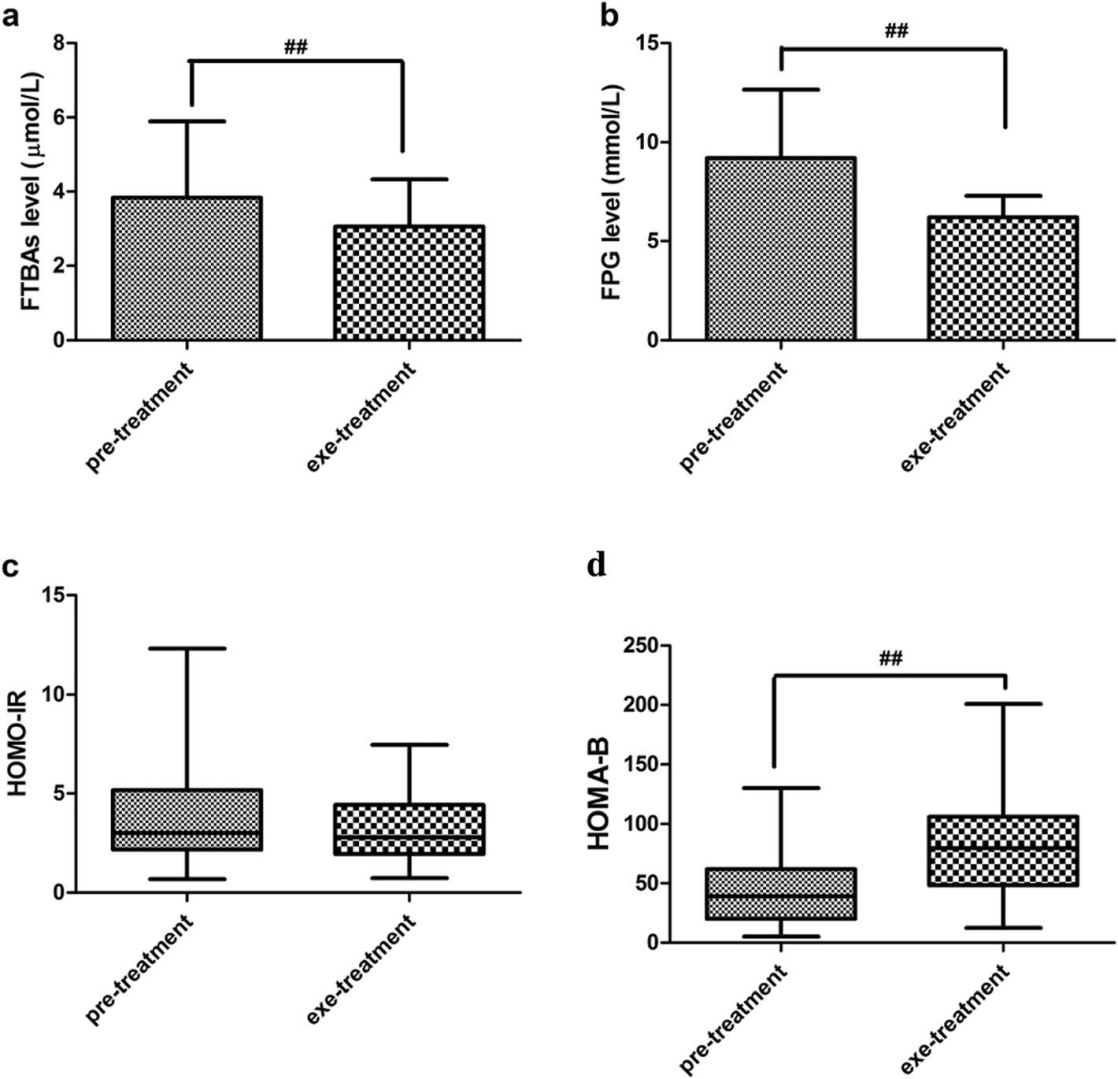
Changes of glycemic parameters after 12-week exenatide treatment for T2DM patients. **a** change of FTBAs; **b** change of FPG; **c** change of HOMA-IR; **d** change of HOMA-B. ^##^*P* < 0.01 vs. pre-treatment

The T2DM patients were given an injection of exenatide for 12 weeks, then changes of FTBAs and other metabolic parameters were measured (Table 2). Weight, BMI, TC and TG levels were significantly decreased after 12-week exenatide treatment compared with pre-treatment. FTBAs were decreased from 3.84 ± 2.06 μmol/L to 3.06 ± 1.27 μmol/L (*P* < 0.01) (Fig. 1a). FPG was decreased from 9.19 ± 3.46 mmol/L to 6.42 ± 1.08 mmol/L (*P* < 0.01) (Fig. 1b). HOMA-IR was decreased from 3.02(2.18–5.16) to 2.80(1.95–4.42), with no statistical significance (Fig. 1c). HOMA-B was increased from 38.81(20.13–61.98) to 79.60(48.47–106.09) (*P* < 0.01) (Fig. 1d).

**Table 2:** Change of parameters after 12-week exenatide treatment in T2DM patients (*n* = 38)

Parameters	Pre-treatment	Exe-treatment	*P*-value*
Weight, kg	93.1 ± 17.1	86.2 ± 18.1	<0.01
BMI, kg/m^2^	31.2(28.5–35.1)	29.4(25.3–32.8)	<0.01
TC, mmol/L	5.05 ± 1.11	4.30 ± 0.92	<0.01
HDL-C, mmol/L	1.15 ± 0.46	1.14 ± 0.28	0.899
LDL-C, mmol/L	2.92 ± 0.80	2.57 ± 0.83	<0.01
TG, mmol/L	2.16(1.26–4.21)	1.37(0.94–2.95)	<0.01
FTBAs, μmol/L	3.84 ± 2.06	3.06 ± 1.27	<0.01
HbA1c, %	9.69 ± 2.02	6.51 ± 0.94	<0.01
FPG, mmol/L	9.19 ± 3.46	6.42 ± 1.08	<0.01
FINS, mU/L	9.44 ± 5.22	11.02 ± 4.53	0.128
C-peptide, mU/L	2.83 ± 1.03	3.11 ± 0.84	0.112
HOMA-B	38.81(20.13–61.98)	79.60(48.47–106.09)	<0.01

Data presented as means ± SD or medians (interquartile range)

*P*-value* were calculated by paired sample t test, exe-treatment vs. pre-treatment

### Correlations between changes of FTBAs and glycemic parameters

**Fig. 2:**
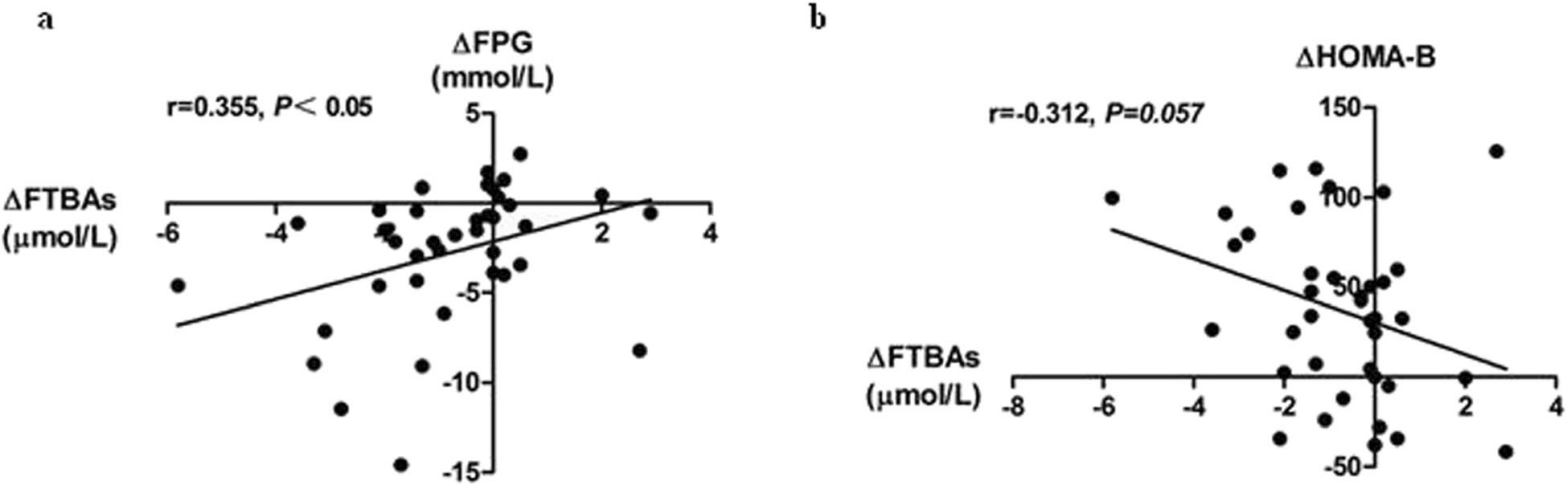
Correlations between changes of FTBAs (ΔFTBAs) and glycemic parameters (ΔFPG, ΔHOMA-B) after 12-week exenatide treatment for T2DM patients. **a** correlation between ΔFTBAs and ΔFPG; **b** correlation between ΔFTBAs and ΔHOMA-B

We conducted a correlation analysis to access the correlation between changes of FTBAs and glycemic parameters. We found that ΔFTBAs was positively correlated with ΔFPG (*r* = 0.355, *P* < 0.05) (Fig. 2a). We did not see statistical significance in correlation between ΔFTBAs and ΔHOMA-B (*r* = − 0.312, *P* = 0.057) (Fig. 2b).

## Discussion

To avoid the interference of metformin and other glucose-lowering drugs on BAs, newly diagnosed T2DM participants without glucose-lowering drugs intake history were recruited. As well known, several drugs may affect the enterohepatic circulation of BAs, namely metformin, inhibitors of the apical sodium-dependent bile acid transporter (ASBT) [18, 19] and bile acids sequestrants (BASs) [20, 21]. Metformin had an effect on BAs reabsorption on intestinal L cells [22]. It may decrease reabsorption of BAs from the intestinal lumen [23] and therefore decrease total serum BAs [24]. On the other hand, metformin was the first-line treatment agent in T2DM patients, so it was necessary to exclude the influence of metformin.

Our results showed that baseline FTBAs of newly diagnosed T2DM patients did not differ from healthy subjects. This was consistent with Andersen E’s study, in which they examined BAs’ kinetics in 15 normal glycemic controls and 22 diet-treated T2DM patients and found no difference [25]. These indicated that diabetic disease status had little influence in BAs, at least BAs in fasting state.

After 12-week exenatide treatment for the overweight newly diagnosed T2DM patients group, FTBAs were decreased from 3.84 ± 2.06 to 3.06 ± 1.27 μmol/L, with a significance of *P*<0.01. The pharmacokinetics of exenatide twice a day are dose proportional, with maximum serum concentrations after a single subcutaneous dose of 2.5 or 5 μg of 56 or 85 pg/mL, respectively, and the area under the concentration–time curve of 159 and 340 pg∙h/mL [12]. Cui YM et al. reported after subcutaneous injection of 2 mg of exenatide weekly in Chinese T2DM patients, that steady state plasma concentrations (299 pg/mL) of exenatide were attained within 8 weeks [26]. It was reasonable to assume that the steady state concentrations of exenatide were almost reached after 12-week injection of exenatide. Previous studies showed that serum BAs were reduced in dipeptidyl peptidase-4 (DPP-4) deficient mice compared to wild type mice, which was explained by a reduction in BAs production and enhanced BAs excretion [27]. Moreover, in rat hepatocyte cultures, both GLP-1 peptide and exenatide reduced CYP7A1, the hepatic cytochrome which converted cholesterol to BAs [27]. Our results and previous researches both suggested not a positive effect of exenatide on biliary physiology in overweight T2DM patients. Nevertheless, in the study of Smits MM et al. 2016, they found that liraglutide increased serum levels of deoxycholic acid in the fasting state and postprandial state, and in faeces [14]. The possible reasons might be: 1) the kind of GLP-1 RA drugs was different, exenatide and liraglutide separately; 2) the population was different, Caucasian and Chinese T2DM patients separately; 3) last but not the least, the baseline of T2DM patients was different. Their recruited patients were treated with a dose of metformin and/or sulfonylurea derivatives for at least 3 months. If the effect of metformin on BAs was big enough, the effect of GLP-1 receptor agonists on BAs could be hardly detected, even though the treatment group had the same baseline of metformin intake with the control group. It was hard to tell what was going on exactly before new valid proofs came out.

Through the correlation analysis, we got the result that the decrease of FTBAs was positively correlated with the improvement of FPG. Accumulating data suggested that BAs improved metabolism through activation of FXR and TGR5 [28–30]. FXR stimulation reduces hepatic gluconeogenesis and improves insulin sensitivity, while TGR5 activation increases energy expenditure and insulin sensitivity. It is quite possible that BAs may have a role in mediating glycemic control. Based on the facts above, our results suggested that the reduction of FTBAs might not exert a positive role in the glycemic control effect of exenatide.

The limitation of our study included: 1) this study was a pilot exploration on exenatide’s biliary effects. More detection about changes of BAs and their metabolites in different prandial phases could reveal more; 2) the sample size was small. More statistical significance might appear if the sample size was enlarged.

## Conclusions

In conclusion, our results demonstrated a decreased FTBAs level after exenatide treatment for 12 weeks, without interference of metformin and other glucose-lowering drugs. The reduction of FTBAs might not exert a positive role in the glycemic control effect of exenatide.

## Data Availability

The datasets used and analyzed during the current study are available from the corresponding author on reasonable request.

## Abbreviations

BAs: Bile acids
FTBAs: Plasma total bile acids in fasting state
GLP-1: Glucagon-like peptide-1
GLP-1 RA: Glucagon-like peptide-1 receptor agonists
T2DM: Type 2 diabetes mellitus
FPG: Fasting plasma glucose
FINS: Fasting serum insulin
HbA1c: Glycated hemoglobin A1c
HOMA-IR: Homoeostasis model assessment for insulin resistance
HOMA-B: Homoeostasis model assessment for β-cell function
BMI: Body mass index
TC: Total cholesterol
LDL-C: Low-density lipoprotein cholesterol
HDL-C: High-density lipoprotein cholesterol
TG: Triglyceride
BAS: Bile acids sequestrants
ASBT: Apical sodium-dependent bile acid transporter
TGR5: Takeda G-protein-coupled receptor 5
FXR: Farnesoid X receptor
